# SLO3: A Conserved Regulator of Sperm Membrane Potential

**DOI:** 10.3390/ijms241311205

**Published:** 2023-07-07

**Authors:** Maximilian D. Lyon, Juan J. Ferreira, Ping Li, Shweta Bhagwat, Alice Butler, Kelsey Anderson, Maria Polo, Celia M. Santi

**Affiliations:** 1 Department of Obstetrics and Gynecology, Washington University in St. Louis, St. Louis, MO 63110, USA; d.lyon@wustl.edu (M.D.L.); juanferreira@wustl.edu (J.J.F.); pli@wustl.edu (P.L.); bhagwat@wustl.edu (S.B.); butlera@wustl.edu (A.B.); kelsey.l.anderson@wustl.edu (K.A.); polo@wustl.edu (M.P.); 2 Department of Neuroscience, Washington University in St. Louis, St. Louis, MO 63110, USA

**Keywords:** membrane hyperpolarization, SLO3, contraception, potassium channels, sperm, acrosomal exocytosis, capacitation, hyperactivated motility, SLO channels, male fertility

## Abstract

Sperm cells must undergo a complex maturation process after ejaculation to be able to fertilize an egg. One component of this maturation is hyperpolarization of the membrane potential to a more negative value. The ion channel responsible for this hyperpolarization, SLO3, was first cloned in 1998, and since then much progress has been made to determine how the channel is regulated and how its function intertwines with various signaling pathways involved in sperm maturation. Although *Slo3* was originally thought to be present only in the sperm of mammals, recent evidence suggests that a primordial form of the gene is more widely expressed in some fish species. *Slo3*, like many reproductive genes, is rapidly evolving with low conservation between closely related species and different regulatory and pharmacological profiles. Despite these differences, SLO3 appears to have a conserved role in regulating sperm membrane potential and driving large changes in response to stimuli. The effect of this hyperpolarization of the membrane potential may vary among mammalian species just as the regulation of the channel does. Recent discoveries have elucidated the role of SLO3 in these processes in human sperm and provided tools to target the channel to affect human fertility.

## 1. Introduction

Sperm have a long and complex maturation process that completes after they are expelled from the body in which they are produced. This post-ejaculatory process gives sperm the capacity to fertilize an oocyte and thus is termed capacitation [1,2]. Capacitation occurs in the female genital tract and involves many molecular changes including increases in cyclic AMP, protein tyrosine phosphorylation [3], intracellular pH [4,5,6,7], potassium ion (K^+^) conductance [8], and intracellular calcium (Ca^2+^) concentration [7,9,10,11,12]. Additionally, the plasma membrane hyperpolarizes to a more negative potential [8,13,14,15,16,17]. These changes culminate in two major physiological changes. The sperm become hyperactive, characterized by an asymmetry of flagellar beating and change in the forces generated [18,19]. This facilitates sperm release from the oviductal reservoir and helps them penetrate through the cumulus and extracellular matrix surrounding the egg (zona pellucida) [20,21]. Additionally, they undergo acrosomal exocytosis, which helps them penetrate the zona pellucida [22,23,24] and exposes binding sites that allow the sperm membrane to fuse with the membrane of the oocyte [25]. Each step of capacitation is required for normal sperm function, but how each step is regulated and regulates other steps has not been fully determined.

A key component of sperm capacitation in many species, from marine invertebrates to mammals, is changes in membrane potential [26,27,28]. Membrane potential is the electrical potential difference (voltage) across a cell’s plasma membrane and is determined by the differences in ion concentrations across the membrane and the selective permeability of the membrane to said ions. One of the most prominent ions for controlling membrane potential in sperm is K^+^. In 1987, K^+^-dependent transient membrane hyperpolarization was first reported in sea urchin sperm in response to a signal from the egg jelly [28]. This hyperpolarization was later shown to also occur in murine and bovine sperm and to be associated with capacitation [13]. Like their mammalian counterparts, human sperm undergo a capacitation-associated hyperpolarization from approximately −40 mV [29] to approximately −58 mV [30].

Several lines of evidence suggest that defects in hyperpolarization can result in infertility. For example, failure to hyperpolarize was correlated with a failure to undergo acrosomal exocytosis in mice [13,27], indicating that sperm membrane hyperpolarization is a key event in sperm capacitation. In humans, electrophysiological studies of patients undergoing in vitro fertilization (IVF) or intra-cytoplasmic sperm injection (ICSI) revealed that ~10% of patients with subfertility have depolarized membrane potentials caused by K^+^ conductance abnormalities [31]. Sperm isolated from men with idiopathic infertility or asthenozoospermia had a significantly more depolarized membrane potential than those from men with normal fertility [32]. Furthermore, capacitated sperm are more hyperpolarized than non-capacitated sperm [26,33]. In 2020, two groups independently used flow cytometry to quantitate membrane potential in sperm from normozoospermic donors and showed that the ability of sperm to hyperpolarize in capacitating conditions correlated with hyperactivation of motility, acrosomal exocytosis, and success in IVF [34,35].

Given the importance of sperm membrane potential in capacitation and fertilization, many researchers have focused on identifying the responsible K^+^ channels. In this review, we describe the evidence of the K^+^ channel SLO3 (KCNU1) regulating sperm membrane potential hyperpolarization during capacitation in several species. Additionally, we highlight several challenges that made it difficult to determine whether or not SLO3 is likewise responsible for hyperpolarization in human sperm. Finally, we present new compelling genetic and pharmacologic data confirming that SLO3 is required for human sperm capacitation and fertility.

## 2. Potassium Channels in Sperm

Sperm from different species are exposed to drastically different environments, from sea water to unique genital tracts. This means that although hyperpolarization is conserved in many species, the mechanisms that drive it must be tailored to the specific environment. One species in which this mechanism has been explored in depth is the mouse. In 1998, the sperm-specific potassium channel SLO3 was cloned by first conducting a low-stringency NCBI Blast query with a cDNA sequence of mouse *Slo1* [36]. A short *Slo3* expressed sequence tag was identified and used to screen a mouse testis cDNA library. Two overlapping cDNAs were isolated and joined to make a complete *Slo3* coding region. A comparison of the SLO1 (KCNMA1) and SLO3 protein sequences showed that the two proteins had similar hydrophilicity profiles, a similar core resembling a voltage-gated K^+^ channel, and long cytoplasmic tail structures. However, the mouse *Slo3* sequence was conspicuously missing two Ca^2+^-binding domains present in the *Slo1* cytoplasmic tail, suggesting that the channel is activated by factors other than Ca^2+^. Instead, the SLO3 channel was found to be activated by intracellular alkalinization.

After the discovery of the SLO3 channel, a potassium current was identified in mouse sperm that shared several key features with the channel [37]. This current, dubbed IKSper, was found to be activated by intracellular pH. The magnitude of the current meant that it was capable of driving large changes in membrane potential [8,37]. These traits matched those of SLO3, and it was confirmed that SLO3 was responsible for IKSper when a SLO3 knockout mouse was generated [37,38,39,40]. Deletion of SLO3 completely abolished the IKSper current. Additionally, sperm from SLO3 knockout mice lack the hyperpolarization that occurs during capacitation. These sperm also lack the resulting Ca^2+^ influx through CatSper, the primary Ca^2+^ channel in sperm that is also necessary for fertility. [38,39]. As a result, SLO3 knockout mice are completely male-infertile.

**Figure 1:**
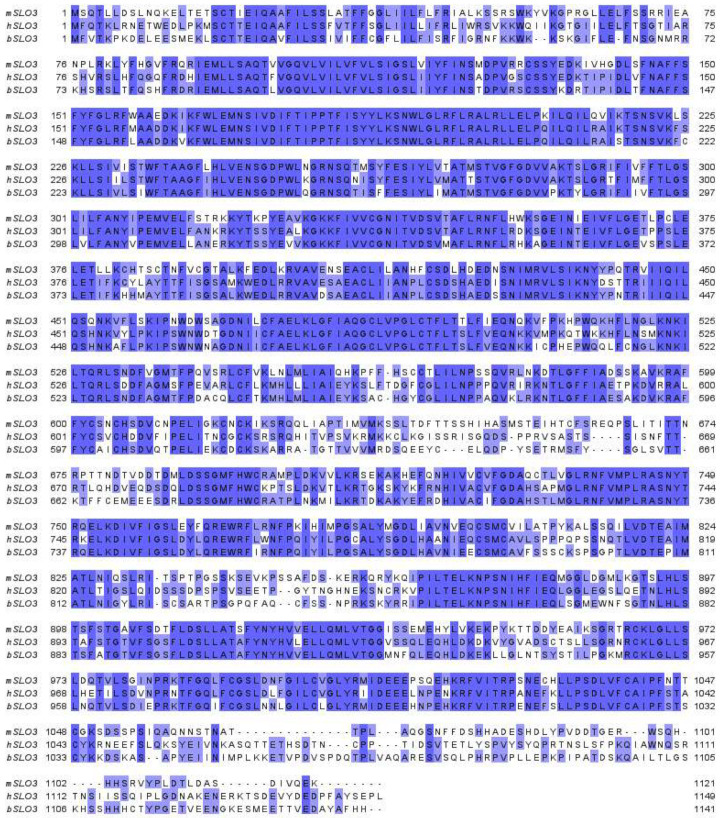
Amino acid sequence homology of mouse (mSLO3), human (hSLO3), and bovine (bSLO3) SLO3. Conserved regions are highlighted in blue. Dark highlighting indicates conservation between three species, light highlighting indicates conservation between two species. Sequence alignment performed using Jalview Version 2 [47,48,49,50].

The *Slo3* gene appears to have arisen by gene duplication of its close paralogue, *Slo1* [41,42,43]. In some non-mammalian animals, such as the spotted gar fish (*Lepisosteus oculatus*) [44] and members of the Salmonidae family, SLO3 has a broad tissue distribution similar to that of SLO1 [45]. However, in mammals, its broad tissue distribution has been lost, and in all mammalian species where it has been studied, including mice, bovines, and humans, SLO3 is only expressed in sperm. Additionally, as is the case with many reproduction-related genes, *Slo3* has evolved rapidly. SLO3 shows only 60% protein sequence identity between mouse, human, and bovine (Figure 1), whereas the SLO1 protein sequence is 90% identical across the same species [46]. Moreover, SLO3 has accumulated species-specific features, which we will discuss later in this review.

## 3. Structure and Gating of the SLO3 Pore-Forming Subunits

**Figure 2:**
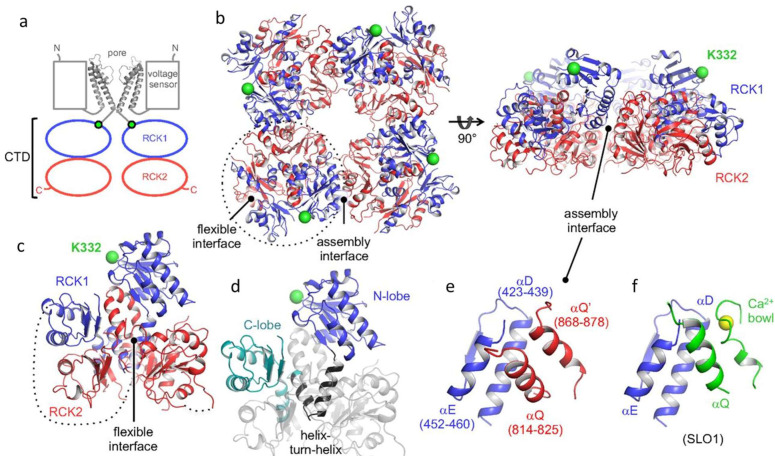
Human SLO3 gating ring structure determined by X-ray crystallography. (**a**) Cartoon of domain topology of two opposing SLO3 α-subunits. (**b**) Crystal structure of the gating ring of a hSLO3 tetramer with RCK1 and RCK2 domains colored in blue and red, respectively. (**c**) A single subunit of the hSLO3 channel and (**d**) highlight of RCK1. (**e**) A closeup of the hSLO3 assembly interface and (**f**) the corresponding region of SLO1 bound to Ca^2+^. The RCK1 N-terminal residue that connects to the transmembrane pore is shown as a green sphere. Ca^2+^ ion is shown as a yellow sphere. Reprinted/adapted with permission from [69].

The pore-forming components of SLO channels are formed by homo-tetramers of α-subunits. Generally, SLO family α-subunits resemble those of voltage-gated K^+^ channels in having transmembrane domains symmetrically arranged around a water-filled, K^+^ selective pore. However, SLO2.1 and SLO2.2 channels have six transmembrane domains (S1–S6) and thus have intracellular N- and C-termini, as is common with members of the voltage-gated K^+^ channel family (Figure 2) [51,52]. In contrast, SLO1 and SLO3 have seven transmembrane domains (S0–S6) and thus have extracellular N-termini.

The cytosolic domains of SLO family channels contain two regulators of K^+^ conductance (RCK) domains, RCK1 and RCK2. These domains sense several intracellular signals and confer each subfamily with distinctive properties [53,54,55,56,57,58]. For example, in SLO1, both RCK1 and RCK2 contain Ca^2+^ sensors [58,59,60]. The “calcium bowl” in RCK2 is composed of a highly conserved string of aspartate residues, which are negatively charged [61]. SLO2.1 and SLO2.2 are also modulated by Na^+^, Cl^−^, and activation of G-protein-coupled receptors [51,52,62,63,64,65]. The cytosolic domain may also be a point of interaction between monomers of different SLO family channels.

The gating of SLO3 is similar to that of SLO1, as the opening of both channels is allosterically regulated by movement of a voltage sensor. This movement is driven by transmembrane potential and conformational change of the cytosolic gating ring induced by intracellular ligand binding. However, there are two important differences between the sensitivities of SLO1 and SLO3 channels to ligands. First, SLO1 is activated by acidification, whereas SLO3 is activated by alkalization [36,66]. Second, SLO1 has several Ca^2+^ binding sites and is activated by a broad range of Ca^2+^ concentrations [58,61,66]. Because of this, SLO1 can function over a broad range of voltages. In contrast, mouse SLO3 is insensitive to calcium and human SLO3 is several orders of magnitude less sensitive to Ca^2+^ than SLO1 [67] and functions in a narrow voltage range near the sperm resting potential.

SLO1 and SLO3 are both sensitive to pH. SLO1 has two histidine residues in the gating ring, which may act as proton sensors and open the channel in response to low intracellular pH [66]. The mechanism of pH modulation of SLO3 is unknown. The half-activation point of SLO3 by pH is estimated to be around 7.7 [68], which is close to the pKa of histidine. With the recently solved structures of the human SLO3 gating ring [69] and complete SLO1 channel [70,71,72], the key residues governing SLO3 regulation by protons should be revealed soon.

## 4. Structure and Function of the SLO3 β and γ Subunits

SLO1 and SLO3 α subunits are associated with several accessory subunits that regulate their expression and biophysical properties [73,74,75]. The α subunit of SLO1 channels is usually associated with auxiliary β- and γ-subunits [76,77,78,79,80], including β1-4 and γ1-2. These auxiliary subunits influence channel pharmacological and gating properties.

An important regulator of SLO3 is γ2, also known as leucine-rich-repeat-containing protein 52 (LRRC52) [81]. This subunit is abundantly expressed in the testis and predominantly interacts with SLO3. It is composed of a single transmembrane segment with an N-terminal extracellular peptide and a short cytoplasmic C-terminal tail [82]. Expression of γ2 depends on SLO3 expression, as *Slo3^−/−^* mice do not express measurable γ2. The primary effect of γ2 is to shift the activation of SLO3 to more negative potentials, as seen in mouse, rat, and human channels [69,82,83]. Importantly, mouse SLO3 currents in the presence of γ2 activate at membrane voltage ranges, similar to those of IKSper [82]. The importance of γ2 for SLO3 activity is evident in *γ2*^−/−^ knockout mice, as IKSper currents in sperm from *γ2*^−/−^ mice activate more slowly and their activation curve is shifted to more positive potentials than the currents from wild-type sperm. Additionally, alkaline pH is less able to hyperpolarize *γ2*^−/−^ sperm than wild-type sperm. As a consequence of all this, the *γ2*^−/−^ male mice are severely subfertile [84], indicating that the γ2 subunit is essential for male fertility.

Mouse and human SLO3 somewhat vary in their responses to γ2. For example, functional expression of human SLO3 requires γ2, and the activation rate and pH sensitivity of human SLO3 channels expressed in *Xenopus* oocytes are increased by γ2. Although mouse SLO3 can be heterologously expressed without γ2, it has increased expression when this subunit is present [67,69,82,85]. Additionally, mouse SLO3 currents display a minor increase in pH sensitivity and activates at more negative potentials [69] when co-expressed with γ2. Nonetheless, γ2 is an important regulator of mouse SLO3. This was shown by expressing mouse SLO3 in *Xenopus* oocytes. Currents obtained from expressing mouse SLO3 alone exhibited a different pH- and voltage dependence than IKSper. However, when co-expressed with γ2, SLO3 produced currents that resembled the native IKSper currents [82].

Unlike γ2, γ1 (LRRC26), γ3 (LRRC55), and γ4 (LRRC38) are minimally expressed in the testis [80,82]. Co-expression of γ1 or γ4 with mouse SLO3 in *Xenopus* oocytes yielded a slight shift towards activation at more negative potentials [82]. Co-expression of γ3 had no effect on mouse or rat SLO3 currents [82]. Thus, γ1, γ3, and γ4 do not appear to play a substantial role in SLO3 regulation.

Because SLO1 is regulated by β subunits, the effects of these subunits on SLO3 have been examined. β1-3 are minimally expressed in the mouse testis and do not appear to functionally regulate SLO3 [78,86,87]. It has even been shown that replacing the mouse SLO1 tail with that of mouse SLO3 ablates the effect of β1 on the channel [88]. Moreover, if co-expressed in Sf9 cells, β1, β3a, and β3b all immunoprecipitate with mouse SLO3 but do not affect the channel gating [87]. β4 is expressed in mouse testis, and co-expression of β4 can increase the surface expression, macro-conductance, and activation kinetics of mouse SLO3 channels in *Xenopus* oocytes [87]. This indicates that only β4 selectively modulates SLO3 expression and function. In humans, β3 and β4 mRNAs are both expressed in the testis [77], but little is known regarding the effects of β subunits on human SLO3.

## 5. Challenges in Determining Whether SLO3 Is Responsible for Human Sperm Hyperpolarization

Both mouse and human sperm undergo hyperpolarization that is required for capacitation. Moreover, mouse and human sperm both have similar K^+^ currents, IKSper and hKSper, respectively. Although SLO3 was conclusively demonstrated to be responsible for mouse sperm hyperpolarization in 2010 [38], four key challenges have impeded our understanding of whether SLO3 is responsible for hyperpolarization in human sperm. These include differences in pH and Ca^2+^ regulation, voltage sensitivity, functional relationships with CatSper, and pharmacology.

### 5.1. Challenge 1: Differences in pH and Ca^2+^ Regulation

Although IKSPer and hKSper have several similarities, these currents have important differences, including that hKSper is less sensitive to pH than is IKSper. Additionally, unlike the IKSper current, the hKSper current is sensitive to intracellular Ca^2+^ [67,89]. Given these reports, Mannowetz et al. suggested that hKSper was conducted by SLO1 rather than by SLO3 [69]. Later, Brenker et al. [67] showed that human SLO3 expressed in *Xenopus* oocytes and Chinese hamster ovary cells is sensitive to both Ca^2+^ and pH. The Ca^2+^ concentrations required to activate SLO3 in both sperm and heterologously expressed in cells were similar (60–1000 µM) and higher than those needed to activate SLO1 [58,67]. Due to these findings, these investigators concluded that SLO3 is likely responsible for the hKSper current.

SLO3 from mice [69,90,91], rats [83], bovines [90], and humans [67] is activated by intracellular alkalinization, though the origin of this pH sensitivity is unresolved. Mouse SLO3 channels are more pH-sensitive than bovine channels and contain two histidine residues in the RCK1 domain, whereas bovine channels have only one histidine [90]. However, substituting one of the histidines in the mouse channel did not significantly alter its pH sensitivity, suggesting that this region is not central to pH regulation. We recently identified a natural structural variant (C382R) of human SLO3 with enhanced pH sensitivity [46]. This variant inserts a positive charge in the -B helix of the N-lobe in RCK1, a critical region likely to interact with the pore-gating region of the channel and may enhance the effectiveness of transduction between the gating ring and the cytoplasmic pore-gating domain. This variant was the third most common single nucleotide polymorphism (SNP) in *Slo3* in the NIH SNP database, possibly indicating positive selection pressure. However, the effect of this SNP on fertility is unknown [46].

### 5.2. Challenge 2: Differences in Voltage Sensitivity

The voltage range of activation of the alpha subunit of SLO3 expressed by itself in heterologous systems varies widely between species. Bovine SLO3 activates at more negative potentials than mouse SLO3. The half-maximal activation of conductance of mouse is at +70–77 mV [36,90] and near +0.5 mV in bovines [90]. In our previous works, we generated a series of chimeric constructs between bovine and mouse α-subunits to map the functional domain responsible for this difference [90]. Our data revealed that the amino acid residues determining this property were distributed over a loop of the RCK1 domain. This region contains the greatest sequence and length polymorphisms among the RCK1 domains of SLO3 channels from many species. This region also has unusually low sequence conservation between bovine and mouse SLO3 channels. Specifically, we found that introducing this region from the bovine channel into the mouse conferred a similar voltage sensitivity as in the bovine channel, but the reverse was not true. This implies that the RCK1 domain of mouse SLO3 is important for voltage sensitivity, but the bovine channel contains other unknown regulatory domains. Expression of the human SLO3 alpha subunit by itself does not result in measurable whole-cell currents. Human SLO3 whole-cell currents can only be obtained in the presence of the human γ2 subunit [67,85,92]. The human SLO3 channel, even in the presence of the γ2 subunit, activates at more positive potentials than mouse or bovine SLO3 in *Xenopus* oocytes [67].

### 5.3. Challenge 3: Differences in Functional Relationship with CatSper

**Figure 3:**
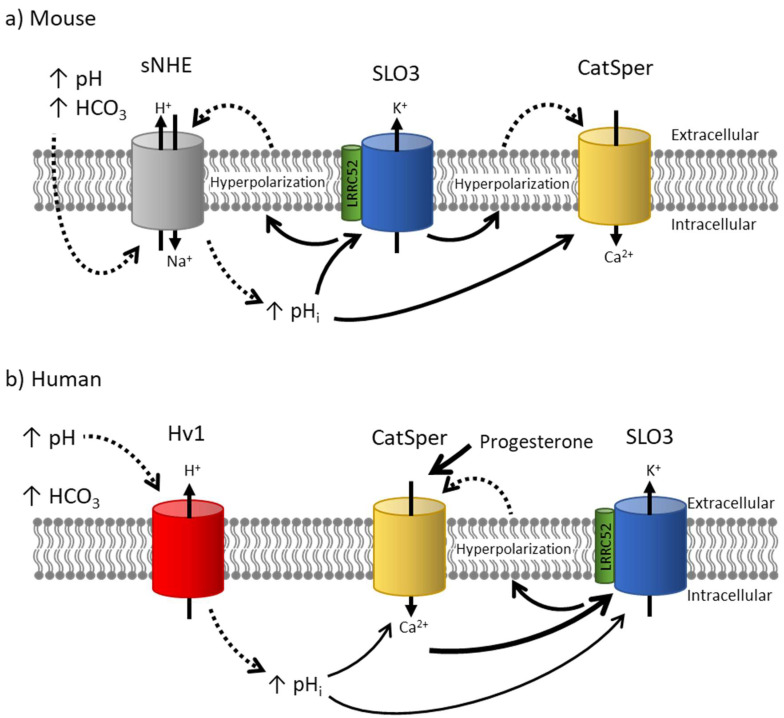
Models of mouse and human SLO3 activity. (**a**) Mouse: The exposure to a more alkaline pH and high [HCO_3_^−^] concentrations in the female tract contribute to an increase in pH_i_, potentially through the activation of the sNHE. This rise in pH_i_ leads to the activation of SLO3 channels, resulting in membrane hyperpolarization. This hyperpolarization enhances calcium influx through CatSper channels, possibly through two distinct mechanisms: Firstly, by increasing the inward driving force of calcium. Secondly, it may further activate sNHE to elevate intracellular pH even more. (**b**) Human: In human sperm, exposure to an elevated external pH could potentially activate the Hv1 channel, resulting in an increase in pH_i_ and contributing to the activation of SLO3 and CatSper channels. However, it is important to note that in humans, SLO3 channels are primarily activated by calcium, while CatSper channels are activated by progesterone. On the other hand, activation of SLO3 leads to membrane hyperpolarization, which has been proposed to remove [Ca^2+^]_i_ oscillations that inhibit CatSper activation. This raises the question of whether SLO3 is activated upstream or downstream of CatSper channels.

In mouse sperm capacitation, the role of SLO3 is extensively characterized (Figure 3). During capacitation, two major ion channels become active, the SLO3 K^+^ channel and the CatSper Ca^2+^ channel. Both channels are essential for fertilization, as knocking out either of them confers male infertility [36,37,38,39,40,93,94,95,96,97]. It is accepted in the field that the increase in intracellular Ca^2+^ necessary to promote hyperactivated motility in the sperm is conducted by CatSper channels [7,37,39,40]. Furthermore, this activation of CatSper seems to be subsequent to and dependent on SLO3 activation, as CatSper activation in *Slo3^−/−^* mice is impaired and can be rescued by alkaline depolarization [11]. A working hypothesis suggests that when sperm are exposed to bicarbonate and an alkaline external pH in the female genital tract, it initially results in an increase in sperm intracellular pH, possibly through the activation of the sperm-specific Na^+^/H^+^ exchanger (sNHE) (unpublished data). This rise in intracellular pH activates SLO3 channels, leading to cell hyperpolarization due to potassium efflux. This hyperpolarization can potentially enhance the driving force and influx of calcium ions through CatSper channels, which exhibit a weak voltage dependence [37]. Additionally, it may further activate sNHE, which contains a putative voltage sensor that could be activated by membrane hyperpolarization [11]. Activation of the sNHE would increase intracellular pH, further stimulating CatSper and SLO3 and establishing a positive feedback loop. Notably, recent results from Chávez et al. in 2020 found more evidence that membrane hyperpolarization induced an increase in intracellular pH of individual sperm [98]. Ultimately, the activation of CatSper channels triggers calcium influx, leading to the necessary elevation in intracellular calcium concentration for sperm hyperactivation.

In human sperm, the temporal sequence of activation of these channels is debated (Figure 3). Because human SLO3 is activated by intracellular Ca^2+^, it was suggested that SLO3 is activated downstream of CatSper in human sperm [99,100]. Contrary to this model, it has been shown that human sperm undergo transient Ca^2+^ oscillations that enact acid extrusion in sperm, inactivate CatSper, and are inhibited by hyperpolarization [101,102]. According to this, SLO3 activity precedes CatSper activity in human sperm as it does in mouse sperm. More experiments need to be conducted to determine the exact sequence of events in human sperm.

The precise details of the SLO3 and CatSper interaction may differ in other ways between mouse and human sperm. For instance, even though sNHE is expressed in human sperm, the proton channel Hv1 has been suggested to have a significant role in the alkalinization of human sperm [103,104]. Hv1 is activated by a combination of the pH gradient and membrane depolarization, and it may also be activated by the removal of zinc after ejaculation and during sperm passage through the female genital tract [103,105]. Activation of this channel could be crucial in raising intracellular sperm pH and subsequently facilitating the activation of CatSper channels. This contrasts with mouse channels, which do not express Hv1 currents and instead rely on sNHE [4,103]. Progesterone at low concentrations activates human CatSper currents. However, it does not have an effect on the mouse currents [67].

### 5.4. Challenge 4: Differences in Pharmacology

Both mouse and human SLO3 exhibit similar responses to certain inhibitors. For example, low concentrations of Ba^2+^ have been found to inhibit mouse SLO3 currents [24,105,106]. This inhibition also extends to hyperpolarization of CHO cells transfected with human SLO3 and human sperm during capacitation [83]. Additionally, high concentrations of TEA (Tetraethylammonium) have comparable effects on both human and mouse SLO3 currents [83,105,106]. However, it is important to note that low concentrations of TEA do not hinder human sperm hyperpolarization during capacitation [83] and have no impact on hKSper currents [65] or human SLO3 in transfected HEK-293 cells [107]. This indicates that, similar to mouse SLO3, human SLO3 is only inhibited by high concentrations of TEA. Another widely used potassium channel blocker, 4-AP (4-Aminopyridine), does not exhibit inhibitory effects on either human or mouse SLO3 when applied externally [108,109]. In contrast, two other inhibitors, quinidine and clofilium, demonstrate strong inhibition of both mouse [105,106,108] and human SLO3 [65,109]. Despite these similarities, mouse and human SLO3 differ in their responses to several other inhibitors.

The first study that examined hKSper currents in human sperm revealed that the current was inhibited by the canonical SLO1 inhibitors charybdotoxin, iberiotoxin, and paxilline [89]. Although later studies confirmed some of these findings, others have presented conflicting results. For example, flow cytometry measurements of membrane potential showed that charybdotoxin and iberiotoxin each strongly inhibited human sperm hyperpolarization during capacitation [26,85]. However, neither drug blocked more than 50% of currents in heterologously expressed human SLO3 currents in CHO cells [85]. Later recordings showed no effect of iberiotoxin on hKSper in sperm [67] or on human SLO3 currents heterologously expressed in HEK cells [106]. These discrepancies may be caused by the different recording conditions used [67,89]. We recently measured the effect of iberiotoxin on human SLO3 expressed in HEK-293 cells and found that it inhibited SLO3 at about 20-fold higher concentrations than SLO1 and had a much wider inhibition curve, making its inhibition of SLO3 currents highly susceptible to different recording conditions [92]. Paxilline, on the other hand, maintained a strong selectivity for SLO1 over human SLO3, as was observed in mice, making it a more useful tool for comparing the inhibition of SLO1 or SLO3 channels across species [92,107].

Both clofilium and quinidine consistently inhibit hKSper currents under physiological conditions [67,108], leading to the prevention of hyperactivation and hyperpolarization of human sperm [67,85,108]. However, determining the extent of SLO3 channel inhibition by quinidine and clofilium in sperm, as well as their impact on sperm physiological properties, is challenging due to the presence of CatSper in these cells [109]. CatSper channels can conduct large currents carried by monovalent cations, which may contribute to the total outward K^+^ current measured in sperm and potentially be mistaken for SLO3 currents. Consequently, quinidine and clofilium might also inhibit CatSper channels [40]. Supporting this notion, Mansell et al. reported that clofilium and quinidine inhibit CatSper channels in human sperm [108]. The off-target effects of these inhibitors on CatSper, as well as their lack of selectivity against other potassium channels, could contribute to their effects on hyperpolarization and hyperactivation. To avoid this confusion, the application of divalent ions, such as 2 mM or higher Ca^2+^, in the external solution can significantly inhibit CatSper from carrying such currents [94,110].

Additionally, studying these channels in heterologous systems can help characterize the inhibitory effects of these drugs on specific channels. For instance, it has been demonstrated that clofilium inhibits human SLO3 currents expressed in CHO cells [26].

Making the situation more complex, inhibitors of CatSper channels can also exert inhibitory effects on SLO3 channels. For example, Mibefradil, a CatSper inhibitor, shows slight and reversible inhibition of mouse SLO3 [111].

Therefore, a dual approach should be employed to characterize the inhibitory effects of these drugs. This includes investigating the inhibitory effects of the drugs on heterologously expressed channels, as well as channels recorded in native cells. Fortunately, such a dual approach is feasible for SLO3 channels, which are well-expressed in both *Xenopus* oocytes and mammalian cell lines. However, achieving expression of CatSper channels in a heterologous system has not yet been accomplished, posing a challenge for their characterization. Summaries of the data on the effects of inhibitors on potassium currents in native cells and in heterologous systems are presented in Table 1 and Table 2, respectively.

## 6. Newly Discovered Variants and Inhibitors Confirm That SLO3 Is Responsible for Human Sperm Hyperpolarization

Two recent papers provide genetic evidence supporting the role of SLO3 in human fertility. Lv et al. reported that a missense mutation and a splice variant of human SLO3 channels are associated with male infertility [96]. However, it should be noted that the male patient in the study presented with asthenozoospermia, a condition characterized by reduced or absent motile sperm [113]. This disorder is not known to be associated with SLO3-deficient mice, as these mice exhibit normal sperm count and motility [38]. Therefore, the presence of this condition suggests that other sperm functional defects unrelated to SLO3 function may have contributed to the infertility observed [96].

In a more compelling case implicating human SLO3 in infertility, a man carrying a missense mutation of the *Slo3* gene (c.1237A>T: Ile413Phe) exhibited sperm that failed to hyperpolarize, undergo acrosome reaction, and achieve successful fertilization in in vitro fertilization (IVF) procedures [97]. However, in intracytoplasmic sperm injection (ICSI), where sperm capacitation is not required, fertilization was successful, as expected for a mutation in the *Slo3* gene. To further confirm the role of SLO3 in this phenotype, the authors generated a mouse line in which the endogenous *Slo3* gene carried the same missense mutation found in the affected men. These mice also exhibited infertility. These findings provide clear evidence that SLO3 is necessary for fertility in both humans and mice and suggest its conserved role in acrosomal exocytosis [97].

We recently described a new inhibitor, VU0546110, which is more than 40-fold selective for human SLO3 over SLO1 [92]. This inhibitor completely inhibited hKSper, confirming that SLO1 channels do not meaningfully contribute to the current. This inhibition also had physiological effects, significantly inhibiting hyperpolarization, hyperactivation, and the acrosome reaction in human sperm. These downstream effects provide further evidence that human SLO3 is necessary for sperm hyperpolarization and fertility.

## 7. Conclusions and Future Directions

The two paralogues *Slo1* and *Slo3* apparently have a common ancestor that underwent a gene duplication event approximately 450 million years ago. Since then, the older gene, *Slo1*, has remained highly conserved in species as diverse as the nematode *C. elegans* worm and *H. sapiens*. Conversely, *Slo3* has been subject to wide evolutionary divergence, functioning in many tissue types in the spotted gar [45] but only functioning in spermatozoa in other species [36]. As a sperm-specific gene in mammals, *Slo3* continues to rapidly evolve to acquire new functional properties, such as Ca^2+^-dependent activation in human sperm [67]. In mammals, SLO3 K^+^ channels hyperpolarize sperm to ensure that Ca^2+^ and possibly also pH reach optimal internal values to drive hyperactivation and regulated acrosomal exocytosis. These actions are essential for sperm to reach and penetrate an oocyte. Because SLO3 plays such an important role in male fertility, it is becoming the focus of studies of possible causes of male infertility and is being targeted to develop new contraceptives.

## Figures and Tables

**Table 1:** Effects of compounds on mouse or human sperm K^+^ currents. Whole-cell patch clamp studies were performed on mouse (IKSper) or human (hKSper) sperm and reported the effect of compounds on potassium currents. Effects were categorized as inhibiting (↓) or having no effect (–).

Compound	Assay	Concentration	Effect	Study
4-AP	hKSper	2 mM	–	[108]
Bupivacaine	hKSper	3 mM	↓	[108]
Charybdotoxin	hKSper, IKSper	1 µM	↓	[89]
Clofilium	hKSper	50 µM–5 mM	↓	[67,108]
Iberiotoxin	hKSper, IKSper	100 nM	–	[67,89]
	hKSper	100 nM	↓	[89]
Lidocaine	hKSper	3 mM	↓	[108]
Paxilline	hKSper, IKSper	100 nM	↓	[89]
Progesterone	hKSper, IKSper	0.5–30 µM	↓	[89,108]
Quinidine	hKSper	300–500 µM	↓	[67,108]
TEA	hKSper	10 mM	–	[67]
VU0546110	hKSper	10 µM	↓	[92]

**Table 2:** Effects of compounds on mouse, human, and rat SLO3 currents expressed in heterologous systems. Studies were examined that measured the effects on whole-cell SLO3 currents in heterologous expression systems of mouse (mSLO3), human (hSLO3), or rat (rSLO3) channels. These data are only for compounds applied externally. Effects were categorized as activating (↑), inhibiting (↓), or having no effect (–).

Compound	Assay	Concentration	Effect	Study
4-AP	hSLO3	25 mM	–	[106]
	rSLO3	100 mM	–	[83]
Ba^2+^	hSLO3	1 mM	↓	[85]
	mSLO3	2 mM	↓	[111,112]
Charybdotoxin	hSLO3	100 nM	↓	[85]
Clofilium	hSLO3	50 µM	–	[85]
	mSLO3	50 µM	↓	[111]
Iberiotoxin	hSLO3	100 nM	–	[85,106]
	hSLO3	0.1–300 nM	↓	[92]
Ketamine	rSLO3	25–500 µM	↓	[83]
LDD175	hSLO3	30 µM	↑	[106]
NS1619	hSLO3	50 µM	↓	[106]
Paxilline	hSLO3	1–30 µM	↓	[92]
Penitrem A	hSLO3	100 nM	↓	[85]
Progesterone	hSLO3	30 µM	↓	[67,85]
Propofol	rSLO3	100–700 µM	↓	[83]
Quinidine	hSLO3	0.1–100 µM	↓	[85,92]
	rSLO3	10–500 µM	↓	[83]
	mSLO3	500 µM	↓	[111]
Slotoxin	hSLO3	100 nM	–	[85]
TEA	hSLO3	20 mM	↓	[106]
	mSLO3	60 mM	↓	[112]
	mSLO3	20 mM	–	[111]
VU0546110	hSLO3	0.3–30 µM	↓	[92]

## Data Availability

No new data were created or analyzed in this study. Data sharing is not applicable to this article.
